# Iranian postmenopausal women’s perspectives on sexual satisfaction: A qualitative study

**DOI:** 10.1371/journal.pone.0326188

**Published:** 2025-07-23

**Authors:** Nasim Shahrahmani, Raheleh Babazadeh, Abbas Ebadi

**Affiliations:** 1 Department of Midwifery, Razi Nursing and Midwifery Faculty, Reproductive and family health research center, Kerman University of Medical Sciences, Kerman, Iran; 2 Nursing and Midwifery Care Research Center, Mashhad University of Medical Sciences, Mashhad, Iran; 3 Department of Midwifery, School of Nursing and Midwifery, Mashhad University of Medical Sciences, Mashhad, Iran; 4 Nursing Care Research Center, Clinical Sciences institute, Baqiyatallah University of Medical Sciences, Tehran, Iran; Babol University of Medical Sciences, IRAN, ISLAMIC REPUBLIC OF

## Abstract

Sexual satisfaction is a key component of human sexuality and is often considered the final stage of the sexual response cycle. Menopause is a physiological phenomenon characterized by various physical, mental, and sexual changes. This qualitative study aimed to explain the concept of sexual satisfaction from the perspective of Iranian postmenopausal women. the study involved 22 married postmenopausal women aged 40–70 years conducted from April 8, 2023, to March 4, 2024, at health centers affiliated with Kerman University of Medical Sciences. Participants were selected through purposive sampling to maximize diversity. Data were collected via semi-structured interviews until saturation was reached and were analyzed following the Graneheim and Lundman method using MAXQDA software. The study identified 16 subcategories and seven main categories include: 1. Sociocultural and religious factors, 2. Individual and contextual characteristics, 3. Psychological factors, 4. Traces of dysfunctional married life, 5. Sexual satisfaction following positive sexual interactions, 6. Sexual helplessness in menopause, 7. Sexual values in menopause. Sexual satisfaction during menopause is a mental, dynamic, and interactive concept significantly influenced by an individual’s perception of their sexual relationship experiences and marital life. Physiological changes during menopause can lead to feelings of sexual helplessness, which impacts sexual satisfaction. Postmenopausal women may find that intimacy and emotional closeness with a sexual partner have a more substantial effect on sexual satisfaction than physical acts. Healthcare providers should develop interventions tailored to the sexual health needs of postmenopausal women, considering cultural, psychological, and physiological factors. Additionally, enhancing access to sexual health education can empower these women to articulate their needs and improve their sexual satisfaction.

## Introduction

Menopause is a physiological event in women’s lives that is accompanied by numerous physical, psychological, and sexual changes, resulting in a complex period. According to the WHO [1], approximately 1/2 billion women over the age of 50 will experience menopause by 2030, with the majority of them (approximately 80%) living in developing countries. The number of postmenopausal women is approximately 3% per year [2]. A study of the average age of menopause in Iran showed that the average age of menopause in Iran is lower than in developed countries, ranging from 46.8 ± 7.8 years, and five percent of those over 55 years old and five percent of those between 40 and 45 experience menopause [3–5]. Menopause can have an impact on various aspects of women’s lives. Among them, sexual performance can be significantly impacted [6]. A study of women aged 40–69 years revealed that 71% were sexually active [7]. Sexual dysfunction is estimated to affect 25% to 63% of all women, with a higher prevalence among postmenopausal women. It ranges between 68% and 86.5% [8].

Sexual satisfaction is one of the components of human sexuality, and it is considered the final stage of the sexual response cycle. Sexual satisfaction is a multidimensional concept that encompasses the emotional and physiological aspects of a sexual relationship. Sexual satisfaction encompasses not only physical pleasure but also all of the positive feelings that follow a sexual relationship [9,10]. In a study by Shikhan et al., examining the level of sexual satisfaction in postmenopausal women, the level of desirable sexual satisfaction was 58.9% [11]. The results of a meta-analysis by Bahari et al. on 3453 Iranian female participants showed that 25% of women had a positive attitude and 58% of women had a neutral attitude towards menopause; also, in general, Iranian menopausal women had poor sexual knowledge [12].

Lawrence and Byers defined sexual satisfaction as an emotional response resulting from individuals’ subjective assessments of the positive and negative aspects of a sexual relationship [13]. Furthermore, observational and experimental studies have shown that sexual satisfaction is associated with higher levels of overall relationship satisfaction [14]. Sex researchers consider sexual satisfaction important for two reasons. First, sexual satisfaction provides a means of assessing relationship partner performance. Second, sexual satisfaction predicts other aspects of the relationship, such as marital quality and stability [15]. A 2013 review study in France revealed that variables and factors such as a) demographic factors as well as physical and mental health status, b) variables related to intimate relationships and sexual response, c) variables related to social support and family relationships, and d) cultural beliefs and values such as religion all play a role in explaining sexual satisfaction. According to the findings of this study, there is a lack of theoretical models to explain sexual satisfaction and the combination of factors influencing sexual satisfaction [9]. According to Schwartz and Young (2009), cases that separate specific elements from the psychological structure are relatively rare in the literature on sexual satisfaction. Regardless of the truth of this issue and the possibility that sexual satisfaction research will continue in the same manner, a comparison of Schwartz’s and Young’s statements with other studies reveals an important point: researchers should not rely on the idea that satisfaction is inherently subjective and that everyone knows what it means [16,17].

Cultural values and norms affect sexual expression and sexual satisfaction; thus, in religious and patriarchal cultures, such as Asian societies, the expression of sexual desires, especially for women, is inappropriate and is considered a threat to the integrity of the family and society, which can be associated with low sexual satisfaction [18]. Sex occurs within a broad social and cultural context, but it is essential that the concept of sexual satisfaction be explained properly in the context in which it occurs. Examining the concept of sexual satisfaction from the perspective of menopausal women is critical for sexual education and sexual therapy because the type and content of education are very different from those before menopause due to menopausal changes [19–21]. The effect of religion on sexual satisfaction varied across studies, with some researchers finding that greater religious beliefs were associated with lower satisfaction in white participants, and other researchers finding no clear difference in sexual satisfaction based on religion [9,22]. In a qualitative study by Merghati-Khoei et al, some religious women considered sexual submission to be a form of submission to divine will. Many women believed that sexual sacrifice would bring them closer to God [23]. In a qualitative study by Bahri et al. (2011), the most important concept that menopausal women expressed about their sexual life was self-sacrifice in the process of managing sexual problems and sexual life. In fact, Iranian menopausal women ignore their sexual needs in order to satisfy their husbands’ sexual needs, which is rooted in their culture and traditional beliefs [24]. As a result, identifying the factors associated with sexual satisfaction is critical for couples seeking to establish and sustain healthy relationships [14].

Postmenopausal women appear to have different understandings of sexual satisfaction. As a result, it is critical to understand the definition of sexual satisfaction for postmenopausal women. Given that women spend nearly one-third of their lives in this period and that this stage of life, like all others, has unique characteristics, this qualitative study was conducted to achieve an in-depth understanding of the concept of sexual satisfaction in the social and cultural context of Iranian women during menopause.

## Materials and methods

Data collection started after Mashhad University of Medical Sciences provided the code of ethics (IR.MUMS.NURSE.REC.1401.039). From April 8, 2023, to March 4, 2024, this qualitative study was conducted in health centers affiliated with Kerman University of Medical Sciences. All menopausal women who had visited Kerman health centers for medical care were included in the research population. Menopausal women between the ages of 40 and 70 years who did not have a known mental illness or limiting physical illness, did not use sex hormones(**such as estrogen and progesterone, libido-enhancing medications and treatments, such as testosterone and various pharmacological agents)**, who could speak Farsi fluently, were willing to share their experiences regarding the research topic, and had engaged in sexual activity with their spouses were the inclusion criteria for this study. The participants were selected via purposive sampling to ensure maximum diversity in terms of age, menopausal age, education, socioeconomic class, and religious beliefs, and the sampling process continued until data saturation. After obtaining the necessary permits at health centers, the researcher explained the study to women who were eligible to participate. If the participants agreed, data were gathered via semi-structured in-depth interviews with open-ended questions. Individual interviews face-to-face were conducted in a quiet room at the health center. Only the participants and the researcher were present in the interview room. The voices of the interviewees were recorded with their permission. The interviews lasted 30–60 minutes. Participants entered the study with informed consent.

The first questions in the interviews were as follows:

- How do you experience sex during menopause??
- How has menopause affected your sex?
- How has sexual satisfaction during menopause changed compared with that before menopause?

What conditions could be effective in increasing your sexual satisfaction after menopause?Wherever necessary, we used probing questions such as“ Can you explain more”?” or “Can you give an example?”.

The data were analyzed via both conventional content analysis and the Graneheim and Lundman methods [25]. In our study, we employed the Graneheim and Lundman method of qualitative content analysis. This theoretical framework is particularly effective for examining nuanced, subjective experiences, especially in sensitive areas like sexual health and satisfaction among postmenopausal women. This approach facilitates a comprehensive understanding of the participants’ perceptions by allowing themes to emerge organically from the data. First, the interviews were transcribed and reviewed several times. The units of analysis were then extracted and, on the basis of the meaning hidden in them, progressed to the abstraction and conceptualization stages before being named with codes. The codes were divided into subcategories, which then categorized based on their similarities and differencesFinally, the main categories were extracted and identified using the most recent concepts from the transcript. If there was any confusion, the transcripts were sent back to the participants for their feedback or corrections and if desired, there was a possibility of feedback from the results to the participants. The 10th version of MAXQDA software was used to manage the qualitative data.

In qualitative research, the concepts of credibility, dependability, confirmability, and transferability are used to ensure trustworthiness [26].The researchers’ long-term involvement with the subject under study, as well as the allocation of adequate time for data collection, ensured the validity of the data. Considering that the researcher had several years of experience working in health centers, had positive interactions with women, and used her professional experiences, she was able to collect accurate and rich data about the research problem. In addition, the researcher checked the data with the rest of the research team. Peer review was another method used to guarantee the consistency of findings (with data reviewed by experts in the field of reproductive health). External reviewers who were not part of the research team but were familiar with qualitative research evaluated the consistency and reliability of the data. Every step of the research process was documented, and a report was created to assess the verifiability of the results. Finally, comprehensive, detailed, and sequential explanations were implemented to enhance the transferability of the data.

## Results

Twenty-two married postmenopausal women participated in the current study. The participants’ average age was 54/5 years and average age of menopause was 49.72yearsTable 1 shows the characteristics of the participated in the current study.

**Table 1:** Details of participants.

Menopause period	Occupation	Number of children	Number of marriages	Education	Duration of marriage	Religious beliefs	Economic situation	Age	Interview order
3	Employed	2	1	PhD	10	Average	Good	50	1
1	Housewife	3	1	Elementary school	30	Average	Average	49	2
10	Retired	2	1	Associate’s degree	27	Low	Average	56	3
3	Housewife	2	1	Elementary school	30	High	Bad	54	4
15	Retired	2	1	PhD	40	Average	Good	67	5
3	Employed	2	1	Master’s degree	25	Average	Good	56	6
3	Housewife	3	1	Diploma	30	Average	Bad	54	7
2	Housewife	2	1	Bachelor’s degree	20	Low	Average	48	8
6	Retired	2	4	Bachelor’s degree	35	Average	Bad	55	9
5	Employed	2	1	Master’s degree	30	High	Good	55	10
1	Housewife	3	1	Diploma	20	Low	Average	47	11
4	Housewife	2	1	illiterate	40	High	Bad	56	12
1	Retired	2	1	Bachelor’s degree	25	Average	Average	47	13
2	Housewife	4	1	Elementary school	41	Average	Bad	52	14
10	Housewife	4	2	Diploma	30	Low	Bad	50	15
14	Housewife	5	1	Elementary school	44	High	Good	62	16
2	Retired	2	1	Master’s degree	39	High	Average	59	17
1	Retired	2	1	Associate’s degree	30	Average	Average	50	18
1	Housewife	6	1	Master’s degree	20	Low	Bad	48	19
2	Retired	1	1	Diploma	13	High	Average	55	20
16	Housewife	0	1	Bachelor’s degree	40	Average	Bad	69	21
10	Housewife	3	1	Associate’s degree	34	High	Good	60	22

During the data analysis and integration process, the Granheim and Lundman method was used to summarize seven main categories, sixteen subcategories and 563 codes as shown in Table 2.

**Table 2:** The primary and secondary themes of Iranian postmenopausal women’s perspectives on sexual satisfaction.

Sub subcategories	Subcategories	Main categories
The educational effect of the family on the interactions of sexual behavior- the negative impact of cultural norms and traditional beliefs- the acquisition of undesirable sexual awareness in society- and the value norms of women in society	**The formation of sexual knowledge in the sociocultural framework of the society**	Sociocultural and religious factors
The negative effect of mother’s concerns on sex- the negative effect of fatigue caused by multiple household and job concerns on sex	**Women’s multiple roles in family and society**	
Being bound by religious requirements regarding sex- false religious beliefs	**Religious beliefs**	
The effects of positive personality traits- The effects of negative personality traits	**Personality characteristics**	Individual and contextual characteristics
Age-Education- Economic factors- Lifestyle- Physical diseases	**Background factors**	
The effect of emotional turmoil in sexual relations- the effect of mental concerns in sexual relations-the effect of relaxation in sexual satisfaction	**Psychological factors**	psychological factors
Depression and stress following menopause- negative sexual self-concept in menopause-psychological distress following lack of orgasm	**Mental disorders in menopause**	
Unfulfilled emotional needs, mutual dissatisfaction in sexual relations, and the acceptance of the current situation	**Emotional injuries**	
Spouse’s sexual dysfunction- lack of sexual flexibility- man’s insufficient sexual knowledge- man’s coercion and sexually harassing behaviors	**Spouse’s sexual dysfunction**	Traces of dysfunctional married life
The negative effects of inability to communicate in sexual relations- the lack of love and sexual intimacy- the lack of sexual conversation; the lack of privacy in joint life- and The inability to create heartwarming moments	**Lack of life and sexual skills in spouse**	
Sexual satisfaction following mutual participation in sexual relations- Understanding the physical and mental changes related to menopause by the spouse- Attention to sexual rights- The necessity of sexual preparation based on foreplay and after play- the proper timing of intercourse	**Sexual satisfaction based on mutual participation in sexual relationships**	Sexual satisfaction following positive sexual interaction
Continuity of sexual performance- Sexual satisfaction as a gift of love and belonging -all round support for the spouse in joint life	**Consequences of positive marital functioning in sexual satisfaction**	
Avoiding sex; unfulfilled sexual expectations- looking at women as a tool	**Sexual boredom**	Sexual helplessness in menopause
Low sexual desire in menopause-decrease or loss of sexual stimulation- decrease or loss of orgasm- pain in menopause- pretense of sexual satisfaction	**Failure to have sex**	
Sexual intercourse consolidates- the couple’s relationship and creates a common identity- sexual satisfaction after confirming -the attractive sexual identity in menopause	**Acceptance of altered sexual attractiveness in menopause**	Sexual values in menopause
The importance of providing emotional satisfaction beyond sexual intercourse in menopause – sexual satisfaction can be achieved by providing mental peace for the spouse	**Reconstruction of emotional intimacy in menopause**	

### Sociocultural and religious factors

The participants’ sexual knowledge developed within the cultural and social context of their society. Factors such as family upbringing, traditional beliefs, the perception that women are sexually submissive despite their reluctance, and the suppression of sexual desires due to cultural taboos all influence sexual interactions. Additionally, societal norms regarding the desirability of female initiation, the need-oriented nature of sexual relationships for men, perceptions of women’s value, and the belief that menopause marks the end of sexual life significantly impact sexual satisfaction.

*One of the people interviewed said*, *“...I do not like to ask my husband for a sexual relationship. I do not know, I mean, I think this request should come from men. I think it is because of our Iranian culture or the influence of my family...” (Participant number 3)*

One of the experiences reported by the participants was a lack of sexual knowledge and the development of undesirable sexual awareness within society, which significantly affected their sexual satisfaction. The participants attributed this lack of knowledge to the absence of educational resources in the past, the circulation of inaccurate sexual information by peers, and the acquisition of unreliable sexual knowledge from social media and the internet.

*One participant expressed, “The incomplete and incorrect teachings of my childhood have had a profoundly negative impact on me. For instance, the first time I heard about sexual relationships, I was taught that it is a sin, shameful, and the behavior of immoral people. This perspective still lingers with me. Whenever I think about these things during intimacy, I feel bad. If those thoughts weren’t with me, I would have felt sexual satisfaction after our encounters” (Participant number 10)*

According to several participants, religious obligations led to lost opportunities for sexual relations. For example, they often felt compelled to bathe after sex, and inadequate bathing conditions prevented them from engaging in sexual activities. Additionally, misconceptions about abstaining from sex during religious days negatively impacted their sexual satisfaction. These restrictions created distress and disrupted couples’ sexual relationships..

*One of the participants stated, “I believe I should take a bath immediately after intercourse to be clean. Now, when it is cold outside and I have to go to work, I avoid my husband, and if we do have sex, I’m not satisfied because of this concern...” (Participant number 10)*

The participants stated that their multiple roles in the family and society, including the burdened responsibilities of motherhood, housekeeping, and work, have caused fatigue and made it difficult or impossible for them to have sex, which has a negative impact on their sexual satisfaction.

*One of the participants said, “You know, the fatigue of work, having children, and daily tasks affect me a lot. I’m truly tired at night, and I’m not truly enjoying and being satisfied as I should...” (Participant number 10).*

### Individual and contextual characteristics

According to the participants’ experiences, the age, education, economic factors, lifestyle, physical diseases, and personality traits of each couple were significant background factors influencing sexual satisfaction. During the interviews with the participants, it was discovered that the importance of sex and the frequency of sex decrease with increasing age, as does couples’ ability to satisfy their sexual desires.

Couples with a high level of education have a better mutual understanding of sexual problems, and as they grow older, they are more likely to learn about proper sexual behavior. Economic problems and inflation also have a significant impact on sexual relationships, so with the rise of economic concerns and mental preoccupation in this area, trying to have a satisfying sexual relationship is no longer a priority in their lives.

*“... My husband and I have enough information to know that sexual desire decreases with age, as do problems with premature ejaculation and vaginal dryness. We understand the conditions and are satisfied with sex under these circumstances...” (Participant number 5).*

Another factor influencing the sexual relationship is each couple’s personality traits, such as introversion and extroversion; being good-natured, violent, moody, shy, miser, strict, and fanatical; having a demanding personality; and the birth order of each couple in their families. In this regard, one of the participants stated:

*“... My husband is a good person, but his language is very harsh, and this makes me distance myself from him, especially in sex; I cannot communicate with him and be satisfied...” (Participant number 13).*

### Psychological factors

Emotional turmoil caused by mental disturbances following bereavement, problems faced by family members, emotional distress caused by family interference and mental concerns such as the presence of children and the spouse’s infidelity all had a negative impact on sex and sexual satisfaction. The participants’ experiences demonstrated the positive effect of relaxation on sexual satisfaction: when they had sex away from anger, mental peace, and stress, they experienced more sexual satisfaction.

*One of the participants stated, “My morals are such that if something happens to my parents or siblings and my mind is preoccupied, my sexual desire decreases, and I do not want him to have sex with me. Even if we do have sex, I do not feel sexually satisfied.” (Participant number 7)*

Mental disorders can occur during menopause. Some of the participants experienced a sense of emptiness and lack after menopause and were upset about it. In addition, negative sexual self-concepts occur during menopause due to a lack of self-confidence and a loss of youthful freshness, as well as physical and mental irritations caused by a lack of orgasm.

*One of the participants said, “I do not have that youthful freshness anymore; my skin is wrinkled, and I like to have sex in the dark...” (Participant number 15).*

Unfulfilled emotional needs caused by a desire not to receive the expected love from one’s spouse, a lack of love and affection from one’s spouse, and a lack of mutual persuasion all contribute to couples’ emotional injuries. Women who compromise with their current situation often experience unsatisfactory sex.

*“I told him many times that I wanted to receive his love throughout the day, and I complained, but it did not help. I got to the point where I said, “Okay, he will not change, but I will, and I accept the situation.” (Participant number 17).*

### Traces of dysfunctional married life

According to the participants’ experiences, male sexual disorders, such as erectile dysfunction, premature ejaculation, low libido, and genital diseases, significantly lead to women’s decreased sexual desire and dissatisfaction.

*One of the participants stated, “It is very important for me to experience complete penetration during sexual intercourse. However, my spouse’s experiences difficulties with both achieving and maintaining an erection, and premature ejaculation. Therefore, the sexual relationship does not satisfy me.” (Participant number 9).*

Deficiencies in life skills, sexual intimacy and affection, open dialog regarding sexual matters, and privacy deprivation are indicators of a dysfunctional marital environment, all of which detrimentally affect sexual satisfaction.

*One of the participants commented, “I would like him to talk to me during sex, but he does not talk. He says I cannot talk to you when we have sex. I would be more satisfied during sex if he talked to me.” (Participant number 7).*

*One participant shared, “I enjoy caressing; it’s not something I dislike. However, since my husband lacks experience and doesn’t know how to do it properly, it dampens my mood. It makes me feel frustrated, and I often tell myself to forget it; I don’t want to engage anymore. If he were more skilled, I believe I would derive satisfaction from our intimacy…” (Participant number. 2).*

### Sexual satisfaction following positive sexual interaction

Sexual satisfaction is achieved through mutual participation in sexual relationships and recognition of each other’s sexual preferences. In addition, many women find themselves satisfied after seeing their spouse’s sense of pleasure. Understanding the physical changes associated with menopause by the spouse, paying attention to each other’s sexual rights, sexual readiness following adequate foreplay and after play, and proper timing of intercourse all contribute to sexual satisfaction through sexual interactions.

*One participant said, “The thing that gives me a great sense of satisfaction is that I like my husband to be sexually active*. *Since I became menopausal, my desire greatly depends on my husband’s behavior. The more he engages in foreplay with me, the more I enjoy it and feel satisfied...” (Participant number 1).*Another effect of positive marital function on sexual satisfaction is the continuation of sexual function, which occurs in the context of maintaining freshness and novelty in interpersonal relationships, sexual attractions, daily attention and affection, daily interactions and conversations, and creating leisure time for two. One of the common experiences among many participants was the positive effect of love in life on sexual satisfaction.

*According to one of the participants, “What is very important to me is to see affection and receive love; we are very much in love with each other, and this has made me satisfied with my sex life...” (Participant number 14).*

According to the participants, spouses’ overall support in their joint life had a significant effect on their sexual satisfaction. Being approved in a joint life, expecting attention and thanks from their spouse, and receiving financial support were all essential to the participants as sex.

*One of the participants said, “It is important to be approved in life; for example, if the food is salty today and he does not complain, it will make me feel better, and if we have sex on that day, it will make me feel more satisfied” (Participant number 13).*

### Sexual helplessness in menopause

Avoiding sexual intercourse entails separating oneself from one’s spouse owing to decreased sexual desire, avoiding sexual intercourse owing to pain during sexual intercourse, and avoiding sexual intercourse owing to decreased secretions and vaginal dryness in menopause.

*One of the participants said, “I am very annoyed during sex because it hurts, but I tolerate it and try to escape from sex...” (Participant number 4).*

Unfulfilled sexual expectations, a lack of satisfaction in sexual relationships, male indifference to female satisfaction, and the use of chilling sentences during relationships all contribute to sexual boredom and dissatisfaction. Additionally, looking at a woman as a tool is one of the factors influencing sexual satisfaction: the fact that the woman is only noticed during sex, as well as the attitude that the wife’s good manners are only displayed when asking for sex, gives the woman a tool look and makes her feel disgusted with sex.

Failure in sexual intercourse is one of the most common experiences of menopausal women, creating a sense of sexual helplessness and having a significant effect on their sexual satisfaction. A large number of participants reported low libido during menopause. Pain and burning during intercourse are among the causes of decreased sexual desire during menopause. Among other changes that occurred during menopause, the participants frequently mentioned a decrease in the importance of sex and pretending to want to have sex despite reluctance, as well as a reduction or loss of sexual stimulation and/orgasm. In this regard, one of the participants stated.

*“Since I experienced menopause, I understand that I have no desire for sex, and My desire for sex has decreased substantially. This is why I usually have sex reluctantly, and it does not satisfy me.” (Participant number 1).*

*One of the participants stated, “Since I went through menopause, I usually have sex two or three times a week, but I do not experience orgasm at least once. I am not satisfied until I reach orgasm...” (Participant number 9).*

Pretending sexual satisfaction in postmenopausal women was frequently mentioned by participants; this pretending to be satisfied occurred as a result of the reduction in sexual stimulation during menopause, not being stimulated despite the partner’s efforts and the difficulty of being satisfied.

*One of the participants said, “Sometimes I’m in a state where I think I’m numb; No matter what my husband does to me, I do not get stimulated or wet. That is why I pretended to reach orgasm.” (Participant number 11).*

### Sexual values in menopause

According to menopausal women’s experiences, the values given to sex during menopause, as well as its outcomes, have a positive effect on sexual satisfaction. Menopausal women believe that sex strengthens the couple’s relationship and contributes to the formation of a shared identity. Many postmenopausal women believe that maintaining their dignity in sexual relationships is critical, and they desire a special position in the minds of their husbands as a result of legal marriage. Furthermore, being attractive and accepted in the eyes of the partner is critical for the continuation of the sexual relationship and, as a result, sexual satisfaction.

*One of the participants said, “Whenever I have a relationship with my husband, my love for him grows. In my opinion, sex brings husband and wife closer together; when people have sex with love, they can be more satisfied.” (Participant number 14).*

One of the important features of rebuilding emotional intimacy during menopause is emotional satisfaction, which is far more important than intercourse. In menopause, women prefer to receive more love, stress, and attention, and emotional satisfaction is more important than sex and even replaces sex. The provision of mental peace by the spouse is very important for a woman, and it requires that a man pay attention to his partner’s mental readiness when asking for a relationship and that this mental peace occurs during the day.

*According to one of the participants, “I tell myself that it is not always supposed to be intercourse; just caressing and touching each other is enough, and it gives me a sense of satisfaction. Since I became menopausal, it’s important for me to feel love and affection; I need to receive love above all else. In my opinion, love and affection are very important for us women, especially when you go through menopause and feel like you’re not the same as before. This gives me a sense of satisfaction…” (Participant number 15).*

## Discussion

The current study aimed to explain the concept of sexual satisfaction from the perspective of Iranian postmenopausal women. Sexual satisfaction during menopause is a subjective, dynamic, interactive, and collaborative concept that is influenced by an individual’s assessment of sexual and marital experiences. The physiological changes associated with menopause cause a sense of sexual helplessness, which has an impact on sexual satisfaction. Sexual satisfaction follows positive sexual interaction, and giving value to emotional and sexual behaviors during menopause is a major factor in achieving sexual satisfaction. Furthermore, the findings of this study revealed that the concept of sexual satisfaction in menopause is a multidimensional, that involves a general self-evaluation of external, internal, and interactive factors. The concept of sexual satisfaction is heavily influenced by underlying factors such as individual and contextual characteristics, socioreligious cultural factors, and psychological variables.

The findings of our research revealed that sexual satisfaction in postmenopausal women is influenced by background factors such as age, education, lifestyle, and underlying diseases. In our study of postmenopausal women, we found that sexual desire, ability to engage in sex, and sexual satisfaction decreased with age. Women with university education experienced fewer sexual problems, leading to greater satisfaction, while employed women or those with an income reported higher levels of sexual satisfaction. Factors such as physical health, lifestyle, premenopausal sexual function, and access to health services also influenced sexual satisfaction during menopause. Although physical health was crucial for active sexual participation and satisfaction, illness did not necessarily preclude the experience of sexual satisfaction. Walfisch (1989) reported that many contextual and individual factors influence women’s sexual satisfaction: age, education, physical health, and personality traits all contribute significantly to women’s sexual satisfaction [27]. In postmenopausal women, increasing age is consistently associated with lower satisfaction [28]. However, not all research has reached the same conclusion. Trompeter’s (2012) study revealed that sexual satisfaction increased with age [29]. Women with a university education have fewer sexual problems and greater sexual satisfaction [18], which could be attributed to their increased awareness of the sexual and physical changes that occur during menopause. Sexual satisfaction decreases as the number of physical diseases increases [28]. Women have higher levels of sexual satisfaction when they live a healthy lifestyle that promotes healthy aging [30]. These findings are consistent with the findings of our study.

Our research findings indicate that women’s sexual desires are strongly influenced by relationships, family perceptions, and their family context, all of which impact sexual satisfaction. Gender taboos in our country stem largely from subcultures that are sensitive about sexual issues. Additionally, the lack of reliable sources for sexual information means that women often rely on inaccurate information from friends and social circles, which can contribute to sexual dissatisfaction. Culture has a significant effect on people’s sexual beliefs, attitudes, and values, as well as their experiences with menopause symptoms, ability to enjoy sexual intercourse, and, as a result, sexual satisfaction or dissatisfaction [23]. Middle-aged and menopausal women may be ashamed in expressing their sexuality due to cultural norms, which inhibit the enjoyment of sex, resulting in an unavoidable decrease in sexual satisfaction caused by factors other than age. Traditional sexual beliefs lead to low levels of sexual satisfaction [17]. There is a significant link between sexual satisfaction and the desire to marry; people who are forced to marry have lower sexual satisfaction [24]. In family-oriented societies, women not only engage in sexual relations to preserve the family but also disregard their sexual desires to achieve this goal [25,26]. These findings are consistent with our findings. The findings of our study revealed that religious sexual rituals, particularly bath rituals, had a dual effect on the sexual satisfaction of the women participating in the current study; thus, while all women adhered to these rituals and considered them a factor in their cleanliness and health, such a requirement occasionally caused distress and disruption in couples’ sexual relationships Sanchez-Fuentes et al. (2014) reported that the effect of religion on sexual satisfaction varied across studies. For example, some researchers discovered that more religious beliefs were associated with lower satisfaction among white participants [9,22,31]. However, women who attend churches frequently report higher levels of sexual satisfaction [32].

Our Research indicates that personality is an emotional, cognitive, and conceptual system that explains an individual’s unique reactions to their environment. Interactions with significant people in the early years instill expectations and beliefs that later shape cognitions and behaviors in romantic relationships during adulthood. Personality is defined as an emotional, cognitive, and conceptual system that explains each person’s reactions to their surroundings. In this context, Besharat (2011) demonstrated that personality traits and characteristics are among the factors that influence men’s and women’s sexual desire. There is a significant relationship between extroversion sexuality, neuroticism, and openness in men and women, implying that sexuality has a negative correlation with neuroticism and a positive correlation with extroversion [33,34], which is consistent with the findings of our sexual satisfaction study.

According to the findings of our research, women complained about the burden of multiple responsibilities, which prevented them from having satisfying sexual relationships. Most families still have a gendered division of labor, but the principle of difference has not been eliminated, and even in two-career families with a working husband and wife, women bear the brunt of household chores. They are expected to handle household chores and child care in addition to full-time employment outside the home [35].

Our research findings indicate that mental health significantly affects sexual satisfaction. Factors such as stress, anxiety, negative sexual self-concept during menopause, unmet emotional needs, and emotional trauma contributed to decreased sexual satisfaction in this demographic. According to Dundon and Rellini (2010), mental health has a greater impact on sexual satisfaction in women over 40 than does physical performance [36]. Sadness, fear, thoughts of failure and separation, frustration and injury, and anticipation are all major causes of sexual dissatisfaction [10]. Depression, anxiety, and stress are all linked to decreased sexual satisfaction [9,37]. Menopause affects women physically, psychologically, and socially. After menopause, women experience a significant decrease in sexual activity and libido, as well as anxiety, as a result of their reluctance [38]. Positive sexual satisfaction is also influenced by body image; in fact, positive body image leads to increased sexual desire and excitability [39]. Women with higher levels of self-confidence and self-acceptance, as well as a greater emphasis on protecting and improving their physical and mental health, have a greater quality of sexual life and sexual satisfaction (44), which is consistent with the findings of our study.

In the present study, the spouse’s sexual function disorders, a lack of life skills, an inability to communicate effectively, a weakness in the sexual conversation, and a lack of ability to create endearing moments and scenes were the main contributors to sexual dissatisfaction. Those who believe that “real” sex necessitates penile-vaginal penetration and ejaculation may resist changing ways and beliefs, leading to dissatisfaction with sex [18]. A lack of knowledge about wives’ menopause and sexual issues affects sexual satisfaction [40]. To make sex more pleasurable, men should engage in behaviors that appeal to women and encourage them to continue having sex [41]. According to Bisson, women are more likely to respond to a relationship’s positive aspects and a reliable partner than they are to let their sexual needs and desires rule them. According to him, the most important aspects of women’s sexual satisfaction are trust, respect, intimacy, and intimate conversation [42]. This was consistent with the findings of our study.

The findings of our research indicate that being attuned to each other’s needs and responding promptly in sexual relationships lead to the fulfillment of expectations and the experience of comfort and sexual satisfaction. This mutual desire fosters a sense of security and connection between partners, enhancing their willingness to maintain closeness and intimacy. Couples expressed that peak sexual pleasure is often reflected in the joy of their partner, making reciprocal sexual experiences more satisfying. Experiences shared by menopausal women suggest that, in fulfilling relationships, there must be an openness to each other’s differences in readiness for sexual relations—whether stemming from varying sexual desires influenced by the menopausal phase or from fatigue and situational readiness that necessitate rest and the postponement of sex. Sexual satisfaction is the result of recognition and mutual understanding in a relationship [43]. People typically check, determine, and judge their level of sex satisfaction with marital satisfaction. It can be argued that initial marital satisfaction is necessary for women to have a fulfilling sexual life [44]. People who are dissatisfied with their interpersonal relationships are less satisfied with sex [45]. Relationship satisfaction is an important predictor of sexual satisfaction. The level of satisfaction that husbands and wives have with their sexual interactions is significantly related to the overall quality of their marital relationships [43]. Social support, defined as an individual’s perception of support and sense of belonging, is correlated with greater sexual satisfaction in women [46]. Social support is linked to increased sexual intercourse and pleasure in postmenopausal women [47]. The level of sexual satisfaction among postmenopausal women is relatively high. Strengthening emotional relationships, increasing intimacy between husbands and wives, increasing self-confidence and physical and mental health, and raising awareness about the menopausal process and the resulting physiological changes make entering this stage of life more enjoyable and result in greater sexual satisfaction [35]. If the couple’s relationship is strong and healthy, most women are willing to have sex whenever their spouses want to have sex, which increases their sexual satisfaction [48]. According to the women in this study, sexual satisfaction during menopause stems from positive emotional and sexual interactions, and sexual satisfaction is one of the outcomes of positive marital function.

This study highlights a significant finding regarding emotional satisfaction in menopausal women that extends beyond sexual intercourse. The results indicate that expressions of affection and caressing outside of sexual activities substantially contribute to these women’s sexual satisfaction. In other words, experiencing loving behaviors from a partner enhances the sense of fulfillment derived from sexual intercourse in menopausal women.Furthermore, emotional satisfaction during this period is not perceived as an independent experience; rather, it is contingent upon receiving attention and affection from a spouse. This attention encompasses loving behaviors, caressing, and physical and sexual intimacy. In this regard, research has also pointed out that sexual activity serves as a motivating force that can lead to close and intimate contact between partners. In menopause, emotional fulfillment is more important than sexual intercourse is [48]. The decrease in the frequency or loss of spontaneity during menopause causes women to prioritize other aspects, such as nonpenetrative sex and valuing quality over quantity [49]. Non genital contact is essential for sexual satisfaction because it heightens the perception of intimacy and emotional closeness [50]. Heyman et al. (2011) examined sexual satisfaction in five countries. The findings revealed that kissing, hugging, and frequently touching one’s partner, as well as caressing and physical intimacy, are important factors in women’s sexual satisfaction. Cuddling is considered a nurturing, nonsexual activity. Women who reported frequent kissing and hugging had a 1.5-percent greater chance of sexual satisfaction than their counterparts did [31]. Menopausal women’s arousal stems from intimacy rather than a desire for physical or sexual stimulation [51]. Emotional responses in sexual relationships include the feeling of pleasure, the level of satisfaction or happiness, and the pleasant feeling [14]. Love and commitment motives in sexual behavior are positively related to sexual satisfaction. Relationship satisfaction is an important predictor of sexual satisfaction [37] The love between couples increases sexual satisfaction [18,45]. Love reflects the quality of sex, [18] which is consistent with our findings.

The experiences of the interviewees in the present study indicate that sexual intercourse that strengthens the relationship and confirms sexual identity in menopause and focuses on sexual rights and the enrichment of interpersonal relationships, motivates sexual intercourse in menopause, and subsequently creates and strengthens the feeling of sexual satisfaction. In this regard, many studies have shown that sexual satisfaction has a positive effect on the mental and physical health of couples and strengthens intimate life and relationships [52,53].Women enjoy being the center of attention of their husbands; therefore, sexual intercourse that begins with the husband expressing affection is usually reported as pleasant. It seems that this type of intercourse creates or strengthens the feeling of being valued in women [54].

The findings of this study showed that Sexual helplessness in menopause and the decline in sexual life during this period are undeniable, which occurs in menopause following low sexual desire in menopause, reduced or lost sexual arousal, reduced or lost orgasm, physical pain and suffering in menopause, and pretending to be sexually satisfied. Hajihasani et al. reported that menopausal women had lower levels of sexual satisfaction than nonmenopausal women did [55]. Less interest, vaginal dryness, and/orgasm problems all play a role in women’s overall sexual satisfaction [56]. Women’s genital fullness and slipperiness, which are important indicators of sexual desire, decrease significantly during menopause [57]. The findings revealed that all postmenopausal women experienced decreased arousal, orgasm, and sexual pleasure. Menopause is particularly associated with increased pain during intercourse and decreased libido [56]. The increasing incidence of dyspareunia with age is a strong predictor of sexual dissatisfaction [58]. These findings are consistent with the findings of the current study and lead to sexual helplessness in postmenopausal women.

One of the limitations of this research was that the fieldwork phase was conducted exclusively in Kerman province, which reduces the generalizability of the study, although four of the participants were not born in Kerman, but had settled in Kerman after marriage. Given the main feature of sexual consent, namely privacy and confidentiality, some participants were not interested in entering into some aspects of it, and the researcher had to do a lot of digging, to reduce the effects of this limitation, the researcher tried to ensure the psychological safety of the participants and interview them in a private environment.

## Conclusion

The concept of sexual satisfaction during menopause is subjective, dynamic, interactive, and collaborative. Sexual satisfaction is heavily influenced by underlying factors such as personal and background characteristics, sociocultural-religious factors, and psychological factors that are influenced by a person’s assessment of sexual relationships and marital life experiences. The physiological changes associated with menopause cause a sense of sexual helplessness, which has an impact on sexual satisfaction. Menopausal women may find that being intimate and emotionally close to a sexual partner is more effective in generating sexual satisfaction than physical sex actions are. Sexual satisfaction among postmenopausal women is influenced by several key factors, as revealed by the participants in this study. Cultural beliefs, religious obligations, and lack of adequate communication with partners were frequently cited barriers that impede fulfilling sexual experiences. Additionally, physical changes associated with menopause and societal perceptions regarding aging and sexuality further contributed to feelings of dissatisfaction. To improve the sexual satisfaction of postmenopausal women, it is essential to implement targeted education and support programs. Health practitioners should provide comprehensive information about sexual satisfaction and the effects of menopause, encouraging open discussions between partners. Workshops and counseling sessions can also promote better communication skills and foster intimacy. Policymakers should consider developing public awareness campaigns that challenge cultural taboos surrounding female sexuality and menopause. Such initiatives could help shift societal attitudes, normalize discussions about sexual health, and empower women to seek support. Additionally, increasing access to healthcare resources specific to the sexual health needs of postmenopausal women will contribute to their overall well-being and relationship satisfaction.
